# Chronic Intermittent Low-Pressure Hypoxia Suppresses Inflammation and Regulates Glycolipids by Modulating Mitochondrial Respiration in db/db Mice

**DOI:** 10.3390/metabo15110707

**Published:** 2025-10-30

**Authors:** Xin Jiang, Keqing Yuan, Xiaofeng Ge, Lili Yu, Yufei Cui, Lianhai Jin, Ying Chang

**Affiliations:** 1 School of Nursing, Jilin Medical University, Jilin 132013, China; 2 School of Basic Medical Sciences, Jilin Medical University, Jilin 132013, China; 3 Department of Medicine and Science in Sports and Exercise, Tohoku University Graduate School of Medicine, Sendai 980-8575, Japan; 4 School of Basic Medical Sciences, Xi’an Jiaotong University, Xi’an 710049, China

**Keywords:** chronic intermittent hypobaric hypoxia (CIHH), type 2 diabetes mellitus (T2DM), insulin resistance, hepatic anti-inflammation, mitochondrial respiratory capacity

## Abstract

**Background/Objectives**: Type 2 Diabetes Mellitus (T2DM) is a chronic disease with persistent hyperglycemia as the main clinical manifestation. Chronic Intermittent Hypobaric Hypoxia (CIHH) is a clinical intervention with intermittent low-pressure hypoxic environmental stimulation. The aim of this study was to evaluate the therapeutic effect and anti-inflammatory effect of CIHH in db/db mice. **Methods**: A simulated 5000 m altitude environment was used to intervene db/db mice. db/db mice were divided into an intervention group (6 h/d) (*n* = 10) and a control group (*n* = 10); meanwhile, healthy mice were divided into two groups, the intervention group (6 h/d) (*n* = 10) and the control group (*n* = 10). The intervention lasted for 6 weeks. Biochemical analyses and pathological tests were performed to evaluate the therapeutic effects, and for the evaluation of mitochondrial respiration, changes in the respiratory capacity of liver mitochondria at various stages of respiration were examined using Oxygraph-2 k. Changes in inflammatory factors in the liver of mice were analyzed using ELISA. **Results**: Following CIHH intervention, db/db mice exhibited significant reductions in body weight, food intake, FBG, TC, TG, and LDL, along with increased HDL levels. Liver indices, PEPCK, G-6-phosphate dehydrogenase, and GLUT2 decreased, while GLUT4 and p-AMPK increased. Hepatic HE staining revealed reduced lipid droplets in the liver. HOMA-IR decreased while HOMA-IS increased. Hepatocyte mitochondrial respiration-related indicators CI + CII stage RCR, CII stage RCR, and CI stage SCR increased, while CI stage SCR decreased. Inflammation-related factors NLRP3, TNF-α, IL-1β, and IL-6 decreased in liver tissue. **Conclusions**: CIHH effectively improves gluconeogenesis and insulin resistance in db/db mice, a result potentially linked to enhanced mitochondrial respiratory capacity and anti-inflammatory effects. Therefore, CIHH offers a potential therapeutic approach for type 2 diabetes.

## 1. Introduction

Type 2 Diabetes Mellitus (T2DM) is a chronic disease with persistent hyperglycemia as its main clinical manifestation [1,2]. This condition affects all organs of the body, and diabetic nephropathy and diabetic cardiomyopathy are the leading causes of disability and death from diabetic complications [3,4]. The pathogenesis of T2DM involves a variety of factors, including inflammation, mitochondrial function, hepatic gluconeogenesis, and insulin resistance [5,6,7]. Current treatment modalities are mainly dietary control and oral hypoglycemic agents. Dietary interventions, though evaluated for their efficacy in T2DM management [8] and oral agents [9], fail to provide complete glycemic control and are often linked to limitations or adverse effects. Given the persistent threat of T2DM to human health [10], new therapeutic modalities are urgently needed.

Chronic intermittent hypoxia (CIHH) is defined as a non-pharmacological intervention in which subjects are exposed to a simulated high-altitude hypoxia environment at fixed times each day for a long period of time and remain in a normal baric environment for the rest of the time [11,12]. At present, many studies have confirmed that CIHH has protective effects on multiple important organs. For example, CIHH alleviates key pathological processes of ischemic cardiac injury, including left ventricular remodeling and myocardial fibrosis [13]; furthermore, it enhances glucose metabolism via upregulating PGC-1α, which contributes to its cardioprotective effects [14]. In the kidneys, CIHH counteracts renovascular hypertension, thereby improving renal perfusion [15]. In the lungs, CIHH prevents the development of pulmonary hypertension by maintaining the homeostasis of endothelial nitric oxide synthase [16]. Beyond the heart, kidney, and lung, CIHH also exerts protective effects, and on skeletal muscles specifically, it attenuates ischemia–reperfusion injury in mouse skeletal muscle [17].

Notably, accumulating evidence supports CIHH-related hypoxic exposure as a beneficial intervention for T2DM. Long-term exposure to high-altitude hypoxic environments has been reported to reduce blood glucose levels [18,19], and population-based studies show a decreasing trend in T2DM incidence among residents living at high altitudes [20,21,22]. Even in high-altitude Chilean populations with a high average body mass index, the prevalence of T2DM remains relatively low [23]. Mechanistically, hypoxia-inducible factors activated by chronic hypoxia can alleviate the changes in renal oxygen metabolism and mitochondrial function caused by diabetes, thereby preventing diabetic nephropathy [24]. Regarding insulin resistance, a phenomenon specific to type 2 diabetes, studies have shown that CIHH may contribute to insulin resistance in mice with type 2 diabetes [11].

Although CIHH can reduce fasting blood glucose (FBG) levels and may improve insulin resistance in type 2 diabetes mellitus (T2DM) models [11,18,22], its specific role in regulating key pathological processes of T2DM—including inflammation and mitochondrial respiration—remains unclear. Inflammation and mitochondrial respiration are not only core drivers of T2DM progression, but also closely intertwined with insulin resistance and hepatic gluconeogenesis. Therefore, examining CIHH’s effects on inflammation and mitochondrial function could elucidate its therapeutic mechanisms in T2DM [5,10]. Therefore, the objective of this study is to evaluate the effects of CIHH on blood glucose levels and insulin resistance in db/db mice (a classic T2DM model). These effects may be attributed to CIHH’s potential to improve hepatic mitochondrial function and exert anti-inflammatory effects. This research provides preliminary foundational evidence for elucidating the regulatory role of CIHH in T2DM-related metabolic disorders.

## 2. Materials and Methods

### 2.1. Animals

Week-old male db/db mice were purchased from Beijing Weishang Lidu Co., Ltd. (Beijing, China), and room temperature was maintained at 23~25 °C. One mouse was kept in each cage on a 12 h light/dark cycle. All experimental procedures were approved by the Ethics Committee for the Use of Animals of Jilin Medical College (2023-KJT-055) and were conducted in accordance with the guidelines for the use of animals.

#### CIHH Treatment

Mice were randomly divided into four groups with 10 mice per group, as follows [25,26]:

- Normal control group: healthy male C57BL/6J mice (no CIHH treatment);
- Model group: male db/db mice (no CIHH treatment);
- Treatment group: male db/db mice with CIHH intervention;
- Positive control group: male C57BL/6J mice with CIHH intervention.

All experimental animals were housed in the experimental chamber. Mice in the treatment group and positive control group were exposed to a simulated low-pressure, low-oxygen environment equivalent to 5000 m above sea level for 42 consecutive days, with daily exposure periods of 6 h (9:00–15:00) [11]. During the remaining time, they were maintained in the chamber’s normal pressure and oxygen environment. The other two groups were housed in the chamber’s normal pressure and oxygen environment throughout the entire study period.

To ensure that the only variable in both the treatment group and the control group was “low-pressure hypoxia” itself and to avoid interference from other environmental factors, the normal control group and the model group were also subjected to the same daily operation procedures (for example, opening the cabin door, checking the environmental monitoring system, and maintaining the same interaction frequency with the mice), but the low-pressure hypoxia program inside the cabin was not activated. This ensures that all groups are exposed to the same level of processing and confinement pressure.

### 2.2. Methods for Index Detection

#### 2.2.1. Histological Analysis

Histological examination of mouse livers was performed after paraffin sectioning. Each 5 μm-thick tissue section was stained with hematoxylin–eosin to visualize tissue structures. To assess the severity of tissue pathology, histopathological features were scored using a standardized scoring system. This assessment was conducted in a double-blind manner by two independent researchers to ensure unbiased evaluation of severity.

#### 2.2.2. Mitochondrial Respiratory Capacity Assay

Using a high-resolution respirometer (Oxygraph-2 k; Oroboros Instruments, Innsbruck, Austria) maintained at 37 °C, we first added tissue suspensions (prepared by clipping 8 mg of tissue and homogenizing it in a glass homogenizer with 10-fold volume of BUFFER) to the Oxygraph-2 k chamber. After adding respiratory substrates, we measured mitochondrial respiration in each state by sequentially adding ADP and inhibitors in the following order: (1) pyruvate (P); (2) glutamate (G); (3) malate (M); (4) ADP (D); (5) cytochrome c (C); (6) succinate (S); (7) FCCP (U); (8) rotenone (ROT); (9) antimycin A (Ama); (10) ascorbate/TMPD. The experimental procedure was strictly followed according to the experimental manual, with the corresponding reagents added in sequence. Oxygen consumption rates were expressed as pmol·s^−1^·islet^−1^. Data was analyzed using DatLab software version 7.0 (Oroboros Instruments). Respiratory control ratios (RCR) were calculated as state 3 respiration divided by state 2 respiration; substrate control ratios (SCR) were calculated as state 3 respiration, with either complex I-linked substrates or complex II-linked substrates divided by state 3 respiration with complex I + II-linked substrates.

#### 2.2.3. Glycolipid-Related Indexes

After 12 h of fasting, blood was collected from the retro-orbital venous plexus of mice and centrifuged; the supernatant was then collected. Mouse blood glucose levels were measured using a glucose assay kit (Huabang Bioengineering, Shanghai, China). Levels of total cholesterol (TC), triglycerides (TG), high-density lipoprotein (HDL), and low-density lipoprotein (LDL) were measured using commercially available ELISA kits (Huabang Bioengineering, Shanghai, China). For the glucose tolerance assay, blood glucose levels in mice were measured at 0, 30, 60, 90, and 120 min after glucose gavage (2 mg/kg).

#### 2.2.4. Insulin Resistance Related Tests

Blood was collected from the venous plexus behind the eyeballs of mice after a 12 h fasting period and centrifuged. The supernatant was collected. Mouse insulin levels were measured using an insulin test kit (Huabang Bioengineering, Shanghai, China). For the insulin tolerance test, insulin (0.01 mL/g) was injected intraperitoneally, and the blood glucose values of each mouse were measured at 0, 30, 60, 90, 120 min after the administration of insulin.

- HOMA-IR = fasting blood glucose (FBG, mmol/L) × fasting insulin (FINS, um/mL)/22.5
- HOMA-IS = 20 × FINS/(FBG-3.5)

#### 2.2.5. Inflammatory-Factor-Related Assays

After liver were minced, homogenate was prepared with a homogenizer at a ratio of 1:10 by weight and volume, and the resulting supernatant was collected. NLRP3, IL-6, IL-1β and TNF-a were measured using the corresponding commercially available ELISA kits (Huabang Bioengineering, Shanghai, China).

#### 2.2.6. Hepatic Gluconeogenesis Related Assay

After liver were minced, homogenate was prepared with a homogenizer at a ratio of 1:10 by weight and volume, and the resulting supernatant was collected. PEPCK, G-6-gase, P-AMPK, GLUT2 and GLUT4 were measured using the corresponding commercially available ELISA kits (Huabang Bioengineering, Shanghai, China).

- Liver index = liver mass (g) × 100/mouse mass (g)

### 2.3. Data Analysis

All data were expressed as means ± standard error of the mean (SEM), and “n” refers to the number of samples for each set of experiments. Statistical analysis was performed by using one-way analysis of variance (ANOVA), followed by Dunnett’s post-test or two-way ANOVA followed by Bonferroni’s post-test or one-sample *t*-test where appropriate. All data were statistically analyzed using GraphPad Prism software version 8. *p* < 0.05 was considered significant.

## 3. Results

### 3.1. Indicators Related to the Improvement of Glycolipid Metabolism in db/db Mice by CIHH

**Figure 1:**
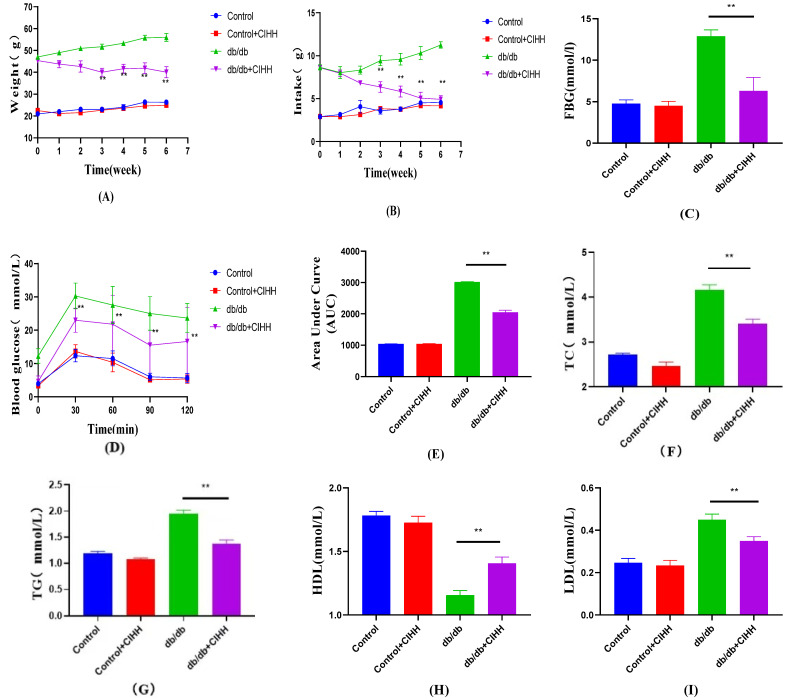
Effect of CIHH on glycolipid-related indices in db/db mice. (**A)** Body weight; (**B**) food intake; (**C**) FBG; (**D**) OGTT; (**E**) AUC; (**F**) TC; (**G**) TG; (**H**) HDL; (**I**) LDL. (*n* = 5) All data are expressed as mean ± SEM and analyzed using one-way analysis of variance (ANOVA) followed by Dunnett’s post hoc test. Differences were considered statistically significant when *p* was ≤ 0.05. ** *p* < 0.01.

The results in Figure 1. showed that after CIHH intervention, body weight, food intake, FBG, TC, TG, and LDL were decreased, and HDL was increased. OGTT, which is one of the most important indices for evaluating the effect of glycemic lowering, showed a significant decrease in its AUC after CIHH intervention. These results indicate that CIHH has a significant improvement effect on glycolipids in db/db mice.

### 3.2. CIHH Ameliorates Hepatic Gluconeogenesis in db/db Mice

**Figure 2:**
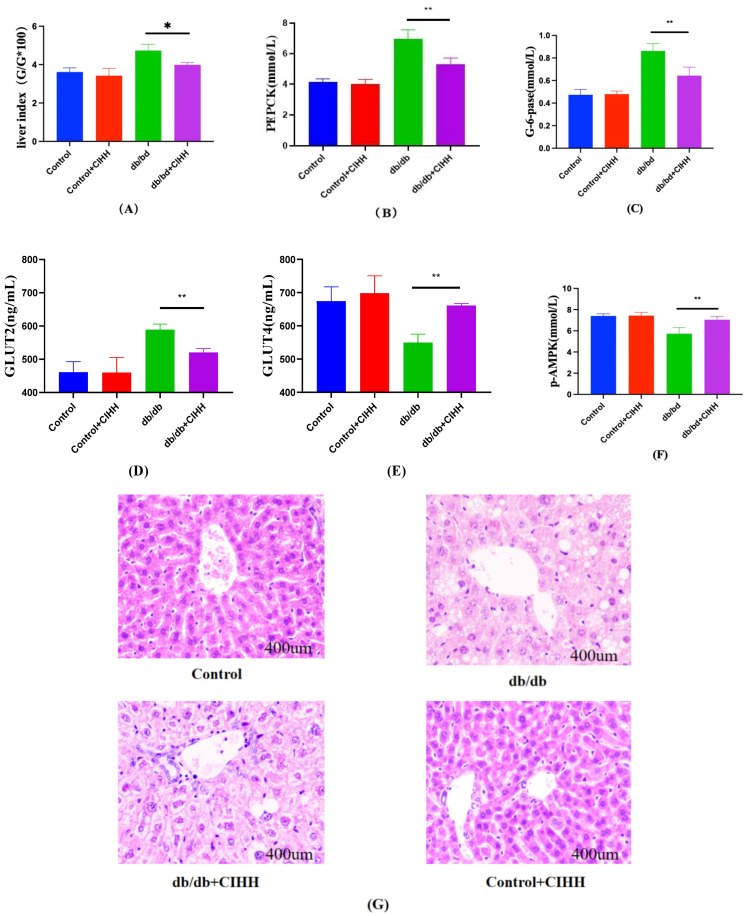
Changes in liver hepatic gluconeogenesis-related indices in db/db mice after CIHH intervention. (**A**) Liver index. (**B**) PEPCK. (**C**) G-6-gase. (**D**) GLUT2. (**E**) GLUT4. (**F**) P-AMPK. (**G**) HE pathology results. (*n* = 5) All data are expressed as mean ± SEM and analyzed using one-way analysis of variance (ANOVA) followed by Dunnett’s post hoc test. Differences were considered statistically significant when *p* was ≤ 0.05. * *p* < 0.05, ** *p* < 0.01.

Results in Figure 2. indicated that CIHH intervention significantly reduced liver index, PEPCK, G-6-PD, and GLUT2 levels in db/db mice, while markedly increasing GLUT4 and p-AMPK levels. Compared to the control group, db/db mice exhibited increased hepatic lipid infiltration, which significantly improved following CIHH intervention. These findings indicate that CIHH intervention improved hepatic tissue structure and partially restored hepatic glucose catabolism function.

### 3.3. CIHH Improves Hepatic Mitochondrial Respiratory Capacity

**Figure 3:**
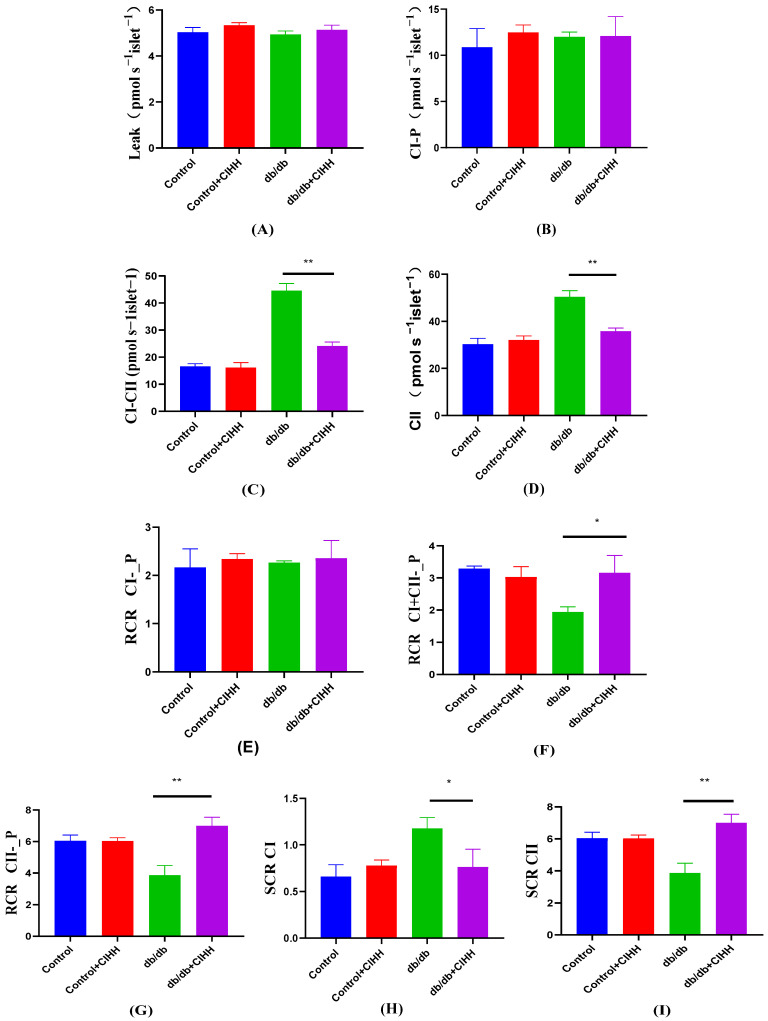
The effect of CIHH on the respiratory capacity of mouse liver mitochondria. (**A**) leak; (**B**) CI-P; (**C**) CI + CII; (**D**) CII; (**E**) CI-P stage RCR; (**F**) CI + CII stage RCR; (**G**) CII stage RCR; (**H**) CI stage SCR; (**I**) CI stage SCR. (*n* = 5) All data are expressed as mean ± SEM and analyzed using one-way analysis of variance (ANOVA) followed by Dunnett’s post hoc test. Differences are considered statistically significant when *p* was ≤ 0.05. *, *p* < 0.05; **, *p* < 0.01. CI, complex I-conjugated substrate; CI + II, complex I + II-conjugated substrate; CII, complex II-conjugated substrate; L, leaky state; P, oxidative phosphorylation.

Results in Figure 3. showed that after CIHH intervention, no significant difference was found in the liver of db/db mice at the Leak and CI_P stages (*p* = 0.2567, *p* = 0.9425), while significant reductions were observed at the CI + CII and CII stages. RCR, an important parameter responding to mitochondrial respiration, was significantly elevated at the CI + CII and CII stages. The substrate control ratio (SCR) of Complex II was significantly higher in the pancreatic islets of db/db mice than that of Complex I. The RCR did not change significantly during the CI phase (*p* = 0.7135) but was elevated during the CI + CII and CII phases after CIHH intervention; the SCR was reduced during the CI phase after CIHH intervention and elevated during the CII phase. These results suggest that CIHH improves mitochondrial respiration in the liver of db/db mice.

### 3.4. Effect of CIHH on Inhibition of Inflammatory Markers in Liver of db/db Mice

**Figure 4:**
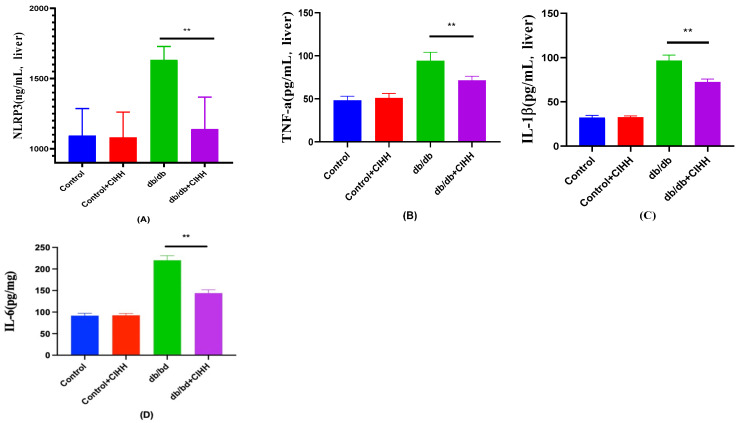
The effect of CIHH on inflammatory markers in the liver of db/db mice. (**A**) NLRP3 in the liver. (**B**) TNF-a in the liver. (**C**) IL-1β in the liver. (**D**) IL-6 in the liver. (*n* = 5) All data are expressed as mean ± SEM and analyzed using one-way analysis of variance (ANOVA) followed by Dunnett’s post hoc test. Differences are considered statistically significant when *p* was ≤ 0.05. **, *p* < 0.01.

According to the results in Figure 4, NLRP3, IL-6,IL-1β and TNF-a were reduced in the liver of db/db mice after 6 weeks of CIHH intervention, and these results suggest that CIHH can reduce inflammatory factors in db/db mice.

### 3.5. CIHH Ameliorates INSULIN Resistance in db/db Mice

**Figure 5:**
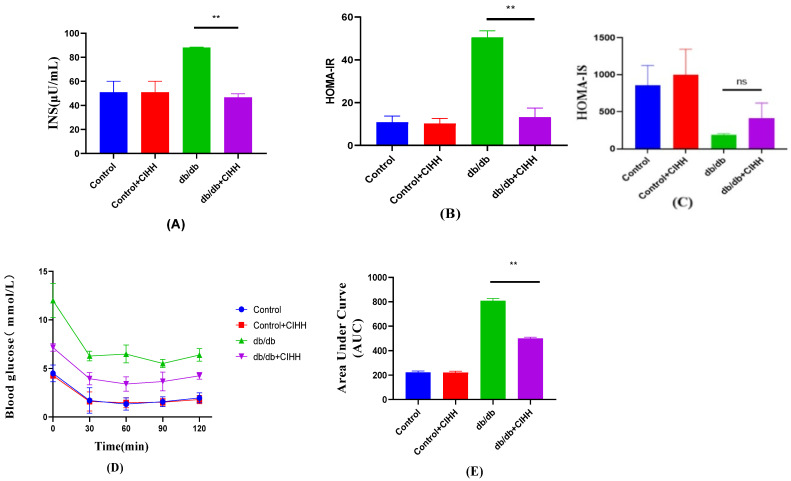
CIHH ameliorates insulin resistance in db/db mice. (**A**) Fasting insulin. (**B**) HOMA-IR. (**C**) HOMA-IS. (**D**) Insulin tolerance assay. (**E**) AUC. (*n* = 5) All data are expressed as mean ± SEM and analyzed using one-way analysis of variance (ANOVA) followed by Dunnett’s post hoc test. Differences are considered statistically significant when *p* was ≤ 0.05. ** *p* < 0.01. ns indicates *p* > 0.05.

The results in Figure 5. showed that insulin tolerance and HOMA-IR were reduced in db/db mice after CIHH intervention. Although HOMA-IS showed no statistical difference in insulin sensitivity between the CIHH intervention groups (*p* = 0.2029), there was a large numerical difference between the two groups. These results suggest that there was an improvement in insulin sensitivity and a decrease in insulin resistance after CIHH intervention.

## 4. Discussion

CIHH demonstrated significant weight reduction effects in db/db mice and exhibited favorable effects in lowering glucose and lipid levels. This study indicates that following CIHH intervention, the oral glucose tolerance test (OGTT) values in db/db mice decreased, demonstrating CIHH’s effective hypoglycemic effects. The accompanying weight loss aligns with clinical treatment goals for type 2 diabetes mellitus (T2DM) [11,15,26,27]. Furthermore, liver histopathology revealed that CIHH did not induce hepatic injury. Pathological examination results confirmed this outcome.

Improved hepatic function in T2DM mice further enhanced the positive effects of reduced glucose and lipid levels [28,29]. Elevated hepatic gluconeogenesis is one of the primary contributors to persistent hyperglycemia in T2DM [30,31,32]. Among glucose metabolism-related molecules, GLUT2 primarily mediates hepatic glucose transport, while GLUT4 facilitates glucose uptake in insulin-sensitive tissues; In T2DM mice, enhanced gluconeogenesis is accompanied by increased GLUT2 expression and decreased GLUT4 expression. This study confirms that CIHH reduces the expression and activity of gluconeogenesis-related enzymes (specifically PEPCK and G-6-Pase), while simultaneously suppressing GLUT2 expression and promoting GLUT4 expression. Furthermore, the upregulation of p-AMPK expression further indicates improved metabolic activity in hepatocytes [33,34,35].

Improving insulin resistance is a core therapeutic goal for T2DM [36,37,38]. This study confirms that CIHH enhances insulin utilization without significantly elevating insulin levels—an effect validated through the Homeostatic Model Assessment of Insulin Resistance (HOMA-IR), Homeoatic Model Assessment of Insulin Sensitivity (HOMA-IS), and insulin tolerance test (ITT) [39,40,41].

Impaired mitochondrial energy production is the primary cause of insulin resistance. In type 2 diabetic patients, the activity of mitochondrial respiratory chain complexes I, III, and IV in the liver is significantly reduced, leading to impaired electron transport, decreased ATP production, and pathological changes in liver tissue [42]. Concurrently, insufficient energy production in hepatocytes reversely inhibits the insulin-mediated translocation of glucose transporters (GLUT4) to the cell membrane, preventing glucose entry into cells and directly inducing insulin resistance [43]. Therefore, mitochondrial function is considered closely associated with type 2 diabetes. This study found that mitochondrial respiration in mouse hepatocytes significantly improved after intervention, suggesting this may be one mechanism by which CIHH ameliorates insulin resistance. The finding that enhanced mitochondrial function improves insulin resistance aligns with studies by Hebert, J. F., Mansouri, A. et al. [44,45]

Research indicates that inflammatory cytokines directly interfere with glucose metabolism. TNF-α and IL-6 suppress the activity of hepatic glycogen synthase (GS), reducing glycogen storage (the liver cannot effectively “store” glucose); IL-1β activates key hepatic gluconeogenic enzymes (e.g., phosphoenolpyruvate carboxylase kinase, PEPCK; glucose-6-phosphatase, G6Pase), promoting conversion of amino acids and lactate into glucose (enhanced gluconeogenesis), leading to elevated fasting blood glucose [46,47,48]. Consequently, elevated inflammatory factors are commonly observed in the livers of type 2 diabetes patients. This study found that CIHH effectively suppressed NLRP3 inflammasome, IL-6, IL-1β, and TNF-α levels. This mechanism likely explains how CIHH improves gluconeogenesis and lowers blood glucose. These findings align with conclusions from studies by Zhao, Q., Jin, J. et al. [49].

This study has certain limitations, as it was conducted exclusively in db/db mice and cannot fully reflect the complexity of human T2DM. This study primarily evaluated the effects of CIHH on the liver, without examining its impact on skeletal muscle, adipose tissue, or the pancreas. CIHH’s influence was assessed solely on mitochondria and inflammation. The effects of CIHH on mechanisms such as HIF signaling or oxidative stress pathways will be systematically investigated in subsequent research. This step-by-step exploration will deepen our understanding of the intrinsic mechanisms underlying CIHH’s hypoglycemic effects.

## 5. Conclusions

CIHH, as a non-pharmacological intervention, demonstrated significant glucose and lipid-lowering effects in a mouse model of type 2 diabetes. Its mechanism of action may be associated with enhanced mitochondrial respiratory function and anti-inflammatory effects. These findings suggest that CIHH holds promise as a potential therapeutic strategy for treating type 2 diabetes.

## Data Availability

The original contributions of this study are included in the article. Further information is available from the corresponding author upon reasonable request.
